# Fate of the Molar Dental Lamina in the Monophyodont Mouse

**DOI:** 10.1371/journal.pone.0127543

**Published:** 2015-05-26

**Authors:** Hana Dosedělová, Jana Dumková, Hervé Lesot, Kristýna Glocová, Michaela Kunová, Abigail S. Tucker, Iva Veselá, Pavel Krejčí, František Tichý, Aleš Hampl, Marcela Buchtová

**Affiliations:** 1 Department of Anatomy, Histology and Embryology, Faculty of Veterinary Medicine, University of Veterinary and Pharmaceutical Sciences, Brno, Czech Republic; 2 Institute of Animal Physiology and Genetics, v.v.i., Academy of Sciences of the Czech Republic, Brno, Czech Republic; 3 Department of Histology and Embryology, Faculty of Medicine, Masaryk University, Brno, Czech Republic; 4 INSERM UMR1109, Team "Osteoarticular and Dental Regenerative NanoMedicine", Université de Strasbourg, Strasbourg, France; 5 Faculté de Chirurgie Dentaire, Université de Strasbourg, Strasbourg, France; 6 Department of Biology, Faculty of Medicine, Masaryk University, Brno, Czech Republic; 7 Department of Craniofacial Development and Stem Cell Biology, King´s College London, London, United Kingdom; 8 Department of Orthodontics, King´s College London Dental Institute, London, United Kingdom; 9 Department of Experimental Biology, Faculty of Science, Masaryk University, Brno, Czech Republic; Team 'Evo-Devo of Vertebrate Dentition', FRANCE

## Abstract

The successional dental lamina (SDL) plays an essential role in the development of replacement teeth in diphyodont and polyphyodont animals. A morphologically similar structure, the rudimental successional dental lamina (RSDL), has been described in monophyodont (only one tooth generation) lizards on the lingual side of the developing functional tooth. This rudimentary lamina regresses, which has been proposed to play a role in preventing the formation of future generations of teeth. A similar rudimentary lingual structure has been reported associated with the first molar in the monophyodont mouse, and we show that this structure is common to all murine molars. Intriguingly, a lingual lamina is also observed on the non-replacing molars of other diphyodont mammals (pig and hedgehog), initially appearing very similar to the successional dental lamina on the replacing teeth. We have analyzed the morphological as well as ultrastructural changes that occur during the development and loss of this molar lamina in the mouse, from its initiation at late embryonic stages to its disappearance at postnatal stages. We show that loss appears to be driven by a reduction in cell proliferation, down-regulation of the progenitor marker Sox2, with only a small number of cells undergoing programmed cell death. The lingual lamina was associated with the dental stalk, a short epithelial connection between the tooth germ and the oral epithelium. The dental stalk remained in contact with the oral epithelium throughout tooth development up to eruption when connective tissue and numerous capillaries progressively invaded the dental stalk. The buccal side of the dental stalk underwent keratinisation and became part of the gingival epithelium, while most of the lingual cells underwent programmed cell death and the tissue directly above the erupting tooth was shed into the oral cavity.

## Introduction

The dental lamina arises as an epithelial thickening along the jaw, which grows deeply into the mesenchyme. Tooth buds are initiated from the dental lamina; however, some exceptions exist as the primary dentition can be initiated very superficially in close proximity to the oral epithelium without being associated with a lamina [1, 2]. In the mouse, the short connection between the tooth and the oral epithelium is called the dental stalk [3–5].

Tooth replacement in vertebrates is initiated from the end of the dental lamina, known as the successional dental lamina (SDL) [2, 6]. This lamina develops after initiation of the first tooth generation. In mammals and reptiles it exceeds the tooth anlagen in the lingual direction, and protrudes more deeply into the mesenchyme, where the second generation bud is formed. The timing of SDL initiation is species-specific and generally arises earlier in dentition with a simpler tooth shape, such as in pythons, in contrast to the complex teeth of mammals [7, 8].

Lifetime and morphology of the dental lamina differ among species depending on how many generations of teeth are initiated during the animal’s life. For example, in diphyodont species such as humans and pigs, the dental lamina starts to disintegrate when the first generation reaches the late bell stage after initiation of the second generation tooth bud [9, 10]. Similarly, in ferret, the SDL loses its connection with the oral epithelium [11]. All previously described species form the second generation of teeth from the SDL, which stays compact, while superficial parts of the lamina become fragmented [12]. Several processes have been described to be involved in lamina disintegration such as cell migration, epithelial-mesenchymal transformation and apoptosis [12]. In contrast to the pig, human and ferret, the dental lamina remains intact in many species of snake and lizard, allowing continuous tooth replacement (polyphyodonty) [1, 7]. In these species, the SDL is very long and located on the lingual side of the tooth anlagen, as in mammalian species [7]. Rudimentary successional laminas that regress have been observed on the lingual side of the first functional teeth in both chameleons and bearded dragons, which like the mouse are monophyodont [13, 14]. Previous observations have suggested that the early degradation of the SDL could play a central role in restricting the number of tooth generations in mammals [12].

Odontogenesis in the mouse starts as an epithelial thickening of the oral epithelium [15]. Consequently, individual tooth germs bud from this thickening into the mesenchyme to initiate individual teeth along the jaw. While the mouse does not form replacement tooth generations, the molars form by a process of serial addition at the back of the mouth [16]. However, a rudimental epithelial protrusion was morphologically recognisable on the lingual side of the dental stalk on transverse sections of mouse (Juuri et al., 2013). Moreover, similar structure was described as attached to human molars where it was previously called as the lateral lamina [17]. Based on the similar morphology and location of this structure it is likely that it could correspond to the successional dental lamina reported in diphyodont mammals. Moreover, the timing of the initiation of this rudimentary structure appears similar to that observed in diphyodont mammals [11, 12]. A similar rudimentary structure was reported in the mandibular first molar of the monophyodont rat and described as a SDL [18]. We therefore decided to investigate this structure further in the mouse, concentrating on the fate of the epithelial cells. We started our SDL analysis just before birth (E16), as the SDL became visible, to assess possible changes in lamina morphology as the developing functional first molar developed from the late bell stage (E16) to eruption (P16). We also focused our attention on a comparison of the dental stalk and the SDL to understand their fates. Furthermore, we tried to understand why the SDL failed to support the development of a further replacement tooth generation and investigated possible mechanisms involved in its regression. Finally, we analysed contingent structural differences between the lingual and buccal sides of the dental stalk in the mouse as differences have previously been described in this structure in other vertebrate species [11, 19].

## Material and Methods

### Animals

Mouse embryos and juveniles (ICR strain) were collected at selected embryonic stages: E16, E18 or postnatal stages: P0, P2, P4, P6, P8, P10, P12, P14 and P16. Mice were obtained from the animal facility at Masaryk university (Brno, Czech Republic). Minipig embryos and fetuses were obtained from Liběchov animal facility (Czech Republic, strain LiM). They were collected between embryonic day 20 and 67 and fixed. Lower and upper jaws were fixed in 4% formaldehyde and then decalcified in 12.5% EDTA in 4% PFA according to the level of tooth mineralisation. All procedures were conducted following the Guide for the Care and Use of Laboratory Animals and a protocol approved by the Animal Science Committee of the Institute of Animal Physiology and Genetics (Czech Republic, approval number: 039/2012 for mice, 020/2010 for pig). Neonatal hedgehog was found in the embryological collection of the University of Veterinary and Pharmaceutical Sciences fixed in 10% formaldehyde.

### Histological analysis

For histological and immunohistochemical analysis, two to three embryos were collected for each analyzed stage. Following decalcification, specimens were washed in tap water and dehydrated in ethanol series and xylene. Samples were embedded in paraffin, and transversal histological sections were prepared (5μm) and stained by Haematoxylin-Eosin. Alternative slides were left unstained for immunohistochemical detection.

### Immunohistochemical analysis

The PCNA staining was used to distinguish the proliferating cells of dental lamina and adjacent oral epithelium during the tooth pre-hatching period. Cytokeratin was used to label intermediate filaments of epithelial tissue, laminin was used to detect basement membrane on the cells and Sox2 was used as a progenitor marker (Table 1).

**Table 1:** List of primary antibodies and pretreatment used for immunohistochemical analysis.

Antibody	Type of antibody	Company and catalogue number	Pretreatment	Dilution of primary antibody
PCNA	mouse—monoclonal	Invitrogen, 931143	Target Retrieval Solution, pH 9 DAKO, cat.n.S2367), 20 min in 97°C water bath	full concentration
Cytokeratin	rabbit—polyclonal	Abcam, ab961	Target Retrieval Solution, pH 9 DAKO, cat.n.S2367), 10–25 min (depending on stage) in 97°C water bath	full concentration
Sox2	rabbit—polyclonal	Cell Signaling, 2748	Trypsin (0.25%) at RT for 10 min; Citrate buffer, pH6, 10mM, 10 min in 97°C water bath	1:50
Laminin	rabbit—polyclonal	DakoCytomation Z 0097	Target Retrieval Solution, pH 9 DAKO, cat.n.S2367), 5 min in 97°C water bath	1:50

Deparaffinised sections were inactivated in endogenous peroxidase by 3% hydrogen peroxide in PBS for 10 minutes at room temperature (RT). Pre-treatment was used depending on the primary antibody (Table 1) in a 97°C water bath. Slides were incubated with primary antibody for 1 hour and secondary antibody for 30 minutes at RT. The peroxidase-conjugated streptavidin–biotin system (Vectastain) and chromogen substrate diaminobenzidine (DAB, Dako, K3466) were used for visualisation of the positive cells or Streptavidin-FITC complex (in the case of laminin). Haematoxylin counterstaining staining enabled negative cell nuclei (blue) to be distinguished from positive (brown) cell nuclei and DAPI was used in the case of fluorescent labelling.

Localisation of apoptotic cells was analysed by the detection of DNA fragments *in situ* by the TUNEL method (ApopTag Peroxidase *in Situ* Apoptosis Detection Kit—S7101, Chemicon, Temecula, USA). Counterstaining with Haematoxylin was performed. The negative control was obtained by omitting the enzyme from the labelling protocol. Sections were photographed under bright field illumination with a Leica compound microscope (DMLB2).

### Mitotic index in the dental stalk area

The dental stalk area together with the rudimental successional lamina was outlined and the number of PCNA-positive and PCNA-negative cells was counted inside the selected area. The percentage of PCNA labeled cells out of the total cell number was calculated. The basal membrane and the edge of the dental stalk connecting to the tooth or the level of oral epithelium served as the morphological references for the outlines (S2 Fig). Morphometric analysis was performed on photos with original magnification 400x to see all details. Ten to fifteen sections were analyzed from each stage (E18, P2, P8). Morphological data were analysed by a one-way ANOVA followed by Tukey´s post-hoc testing in Statistica 6.0 (StatSoft, Czech) to determine possible differences in proliferation activity during development. The significance level was set at p < 0.05.

### 3D reconstruction

The dental stalk of E18, P2 and P6 mouse embryos (one embryo for each stage) were selected for the analysis of RSDL structure. Serial transverse sections were photographed and 3D image reconstruction was performed using WinSurf software (version 4.3, developed by Scott Lozanoff, University of Hawaii). The dental stalk area including RSDL structure and part of enamel organ was outlined to see relationships among structures.

### Transmission electron microscopy

The dental laminas of E18, P2, P8 and P14 mouse embryos (three to four embryos for each stage) were fixed in 3% glutaraldehyde-0.1M cacodylate buffer for 2 hours. After three washing steps in 0.1M cacodylate buffer, samples were post-fixed in 1% OsO_4_ solution in the same buffer for 1 hour. Then, specimens were dehydrated in ethanol and acetone and embedded in epoxy resin Durcupan. Semi-thin sections were stained with toluidine blue. Ultrathin sections were contrasted with uranyl acetate and lead citrate solutions. Samples were analysed in Morgagni 268 TEM (FEI Company) and pictures were taken with a Veleta CCD Camera (Olympus).

## Results

### Rudimental successional dental lamina was initiated in monophyodont mouse

**Fig 1:**
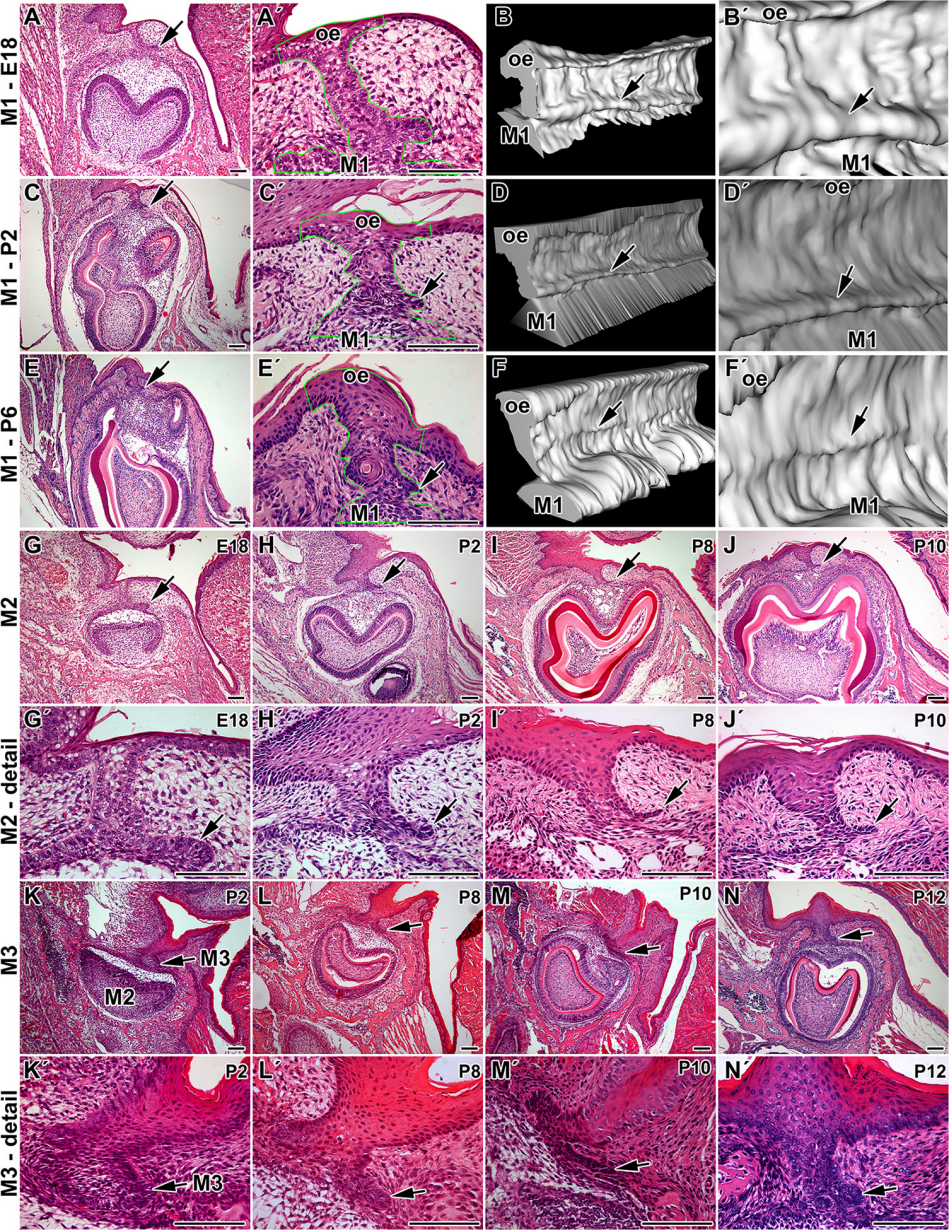
Dental lamina morphology in monophyodont mouse. **A:** Dental lamina in monophyodont mouse at E18 is composed of the dental stalk area (ds) connecting the first lower molar (M1) to the oral epithelium and rudimental successional dental lamina (arrow). Dental stalk is very short and tooth develops in the close proximity to the oral epithelium. **A´:** Detail of dental stalk area at E18. **B:** 3D reconstruction of the dental stalk area as inclined view from rostral part. **B´:** Detail of continuous rudimental successional dental lamina (arrow) at E18. **C:** Lower power of the first molar and detail of the dental stalk area (**C´**) at P2 with smaller successional dental lamina. **D, D´:** Rudimental successional dental lamina (arrow) is smaller at P2 especially at rostral and caudal end of the first molar (M1) as shown by 3D reconstruction. **E, E´:** Dental stalk area at P6. **F, F´:** The size of successional dental lamina is much smaller at P6 and it forms distinct structure just at cusps level (arrow). **G, G´:** The successional lamina is protruding on the lingual aspect of the second molar (M2) at E18. **H, H´:** The rudimental successional dental lamina of M2 became smaller at P2 and P8 (**I, I´**). **J, J´:** In contrast to the first molar area, the lamina is still visible close M2 at P10. **K, K´:** There is no successional lamina close the third molar at P2 as the teeth reached just early cup stage. **L, L´:** In the area of the third molar (M3), successional lamina was well visible at P8. **M, M´:** Later in development became thinner. **N, N´:** At P12, it was still observable at P12 in contrast to the first and the second molar area. arrow—rudimental successional dental lamina, oe—oral epithelium. Hematoxylin-Eosin. Scale bar—100 μm

A rudimental successional lamina (RSDL) was visible on the lingual side of the first lower molar during late embryonic and early postnatal stages (Fig 1). It has appeared as epithelial protrusion of the dental stalk just above the enamel organ (Fig 1A and 1A´). The RSDL was continuous along the first molar as shown by 3D reconstruction (Fig 1B and 1B´); however, this structure became smaller and less distinct during postnatal stages (Fig 1C, 1C´, 1D and 1D´). At P6, the structure of RSDL was more distinct above cusps while between cusps or at beginning of the molar was just discrete (Fig 1E, 1E´, 1F and 1F´). Same rudimental lamina was found attached to the second and third lower molars (Fig 1G–1N). The initiation and expansion of RSDL was delayed accordingly to postponed development of caudal teeth. While RSDL was continuous in the teeth area, this structure completely disappeared in the area between lower molars (S1A, S1D and S1G Fig).

Same epithelial structure but with smaller size was found to be attached to the molar at the upper jaw at embryonic as well as postnatal stages (S2 Fig).

As we observed RSDL in all analyzed molars in the upper as well as lower jaw, we proceed next more detail analysis just including the first molar in the lower jaw.

### Ultrastructural analysis revealed signs of RSDL degradation during postnatal stages

**Fig 2:**
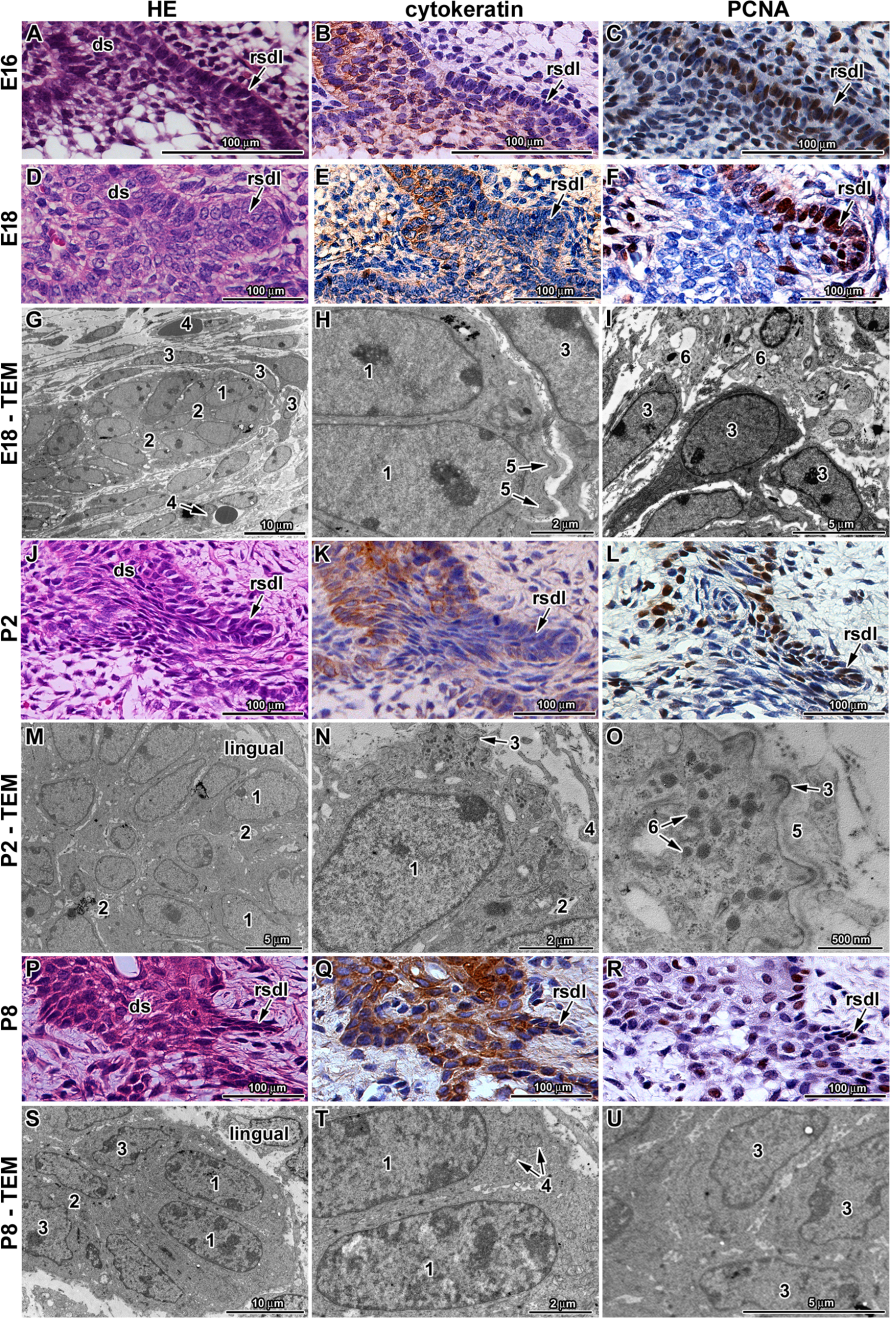
Rudimental successional dental lamina in the mouse. **A-C:** The successional dental lamina is visible from late embryonic stages (E16) as the epithelial thickening on the lingual side of the outer enamel epithelium. **B:** Cytokeratin-positive cells are located mostly in the dental stalk area while epithelial thickening is less positive. **C:** PCNA-positive cells are located in the basal epithelial layer with higher appearance on the lingual side and in the epithelial thickening. **D-F:** Later in development at E18, the RSDL becomes separated from the tooth germ and forms large epithelial protrusion. The area of the RSDL is cytokeratin-negative **(E)** and PCNA-positive **(F). G-I:** Ultrastructure of the RSDL at E18 shows the evidence of several layers of epithelial cells (1) with reduced intercellular spaces (2). Flat mesenchymal cells surround the tip of the lamina (3) together with small blood vessels (4). Folds of the epithelial cells (5) are located on the tip of the lamina. Several macrophages (6) enclose the RSDL area. **J-L:** During early postnatal stage (P2), the successional lamina is still well distinguishable epithelial structure. The area of the RSDL is cytokeratin-negative **(K)** and PCNA-positive **(L)**. **M-O:** Ultrastructure of the RSDL at P2 reveals several layers of epithelial cells with smooth nuclear contours (1) and small intercellular spaces (2). Large folds of epithelial cells are located on the tip of the successional dental lamina (3) containing numerous lysosomes (5). Fibroblasts (4) exhibit cytoplasmatic processes closely to basement membrane surrounded by large amount of extracellular matrix (5). **P-R:** Later in development, the lamina forms just rudimental thin epithelial projection. The area of the RSDL is cytokeratin-positive (Q) and only few PCNA-positive cells are located in this area (R). **S-U:** Ultrastructure of the RSDL at P8. Only leading epithelial cells contain nucleus with smooth contours (1) with a few organelles—mostly mitochondria (4). Other cells exhibit undulated nuclear envelope (3). Intercellular spaces between epithelial cells are still reduced (2). Proliferating cells are labeled by PCNA antibody (brown nuclei, arrows). Epithelial cells are labeled by cytokeratin antibody (brown cytoplasm). Negative cells are counterstained by Hematoxylin (blue nuclei).

**Fig 3:**
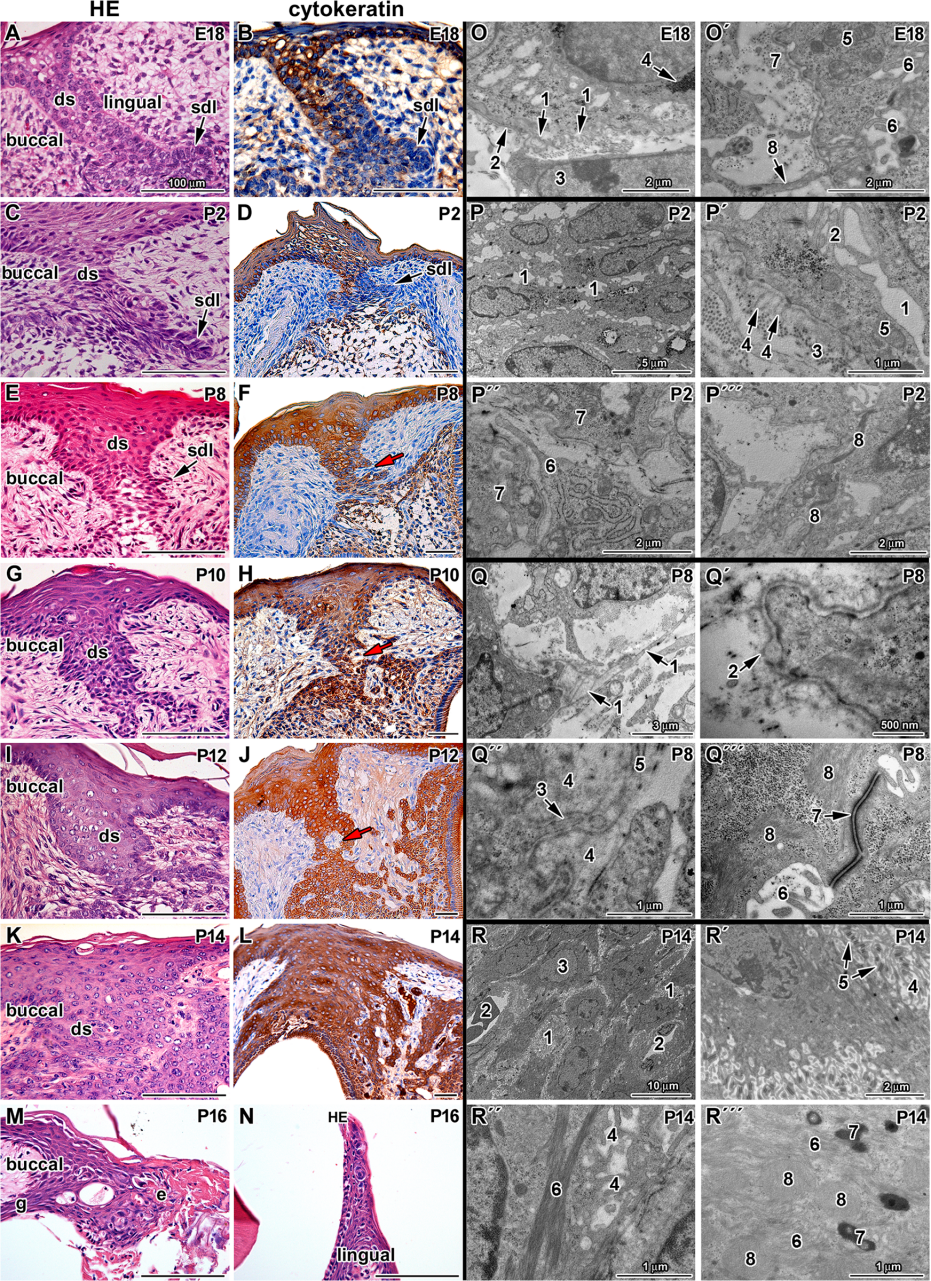
Morphogenesis of the dental stalk area in mouse. Transversal sections through the dental stalk area of the first molar in the lower jaw of mouse. **A-D:** The dental stalk epithelium (ds) is compact at early developmental stages (E18, P2). **E-H:** Later in development, small bays of mesenchyme (red arrow) with capillaries disrupt epithelial tissues (P8, P10). **I-L:** Just before molar eruption, epithelial tissues integrity is disrupted by connective tissue islands (P12 and P14). Degradation of epithelium is obvious during time of eruption (P14 and P16). **M,N:** At P16, the buccal area of the dental stalk becomes the part of gingiva (g) and area above erupting teeth degrades and deceased tissue is peeled off the epithelium (e). ds—dental stalk, sdl—successional dental lamina. Red arrow—bays of connective tissue protruding into the epithelium. HE—Hematoxylin-Eosin. Epithelial cells are labeled by cytokeratin antibody (brown). Negative cells are counterstained by Hematoxylin (blue nuclei). Scale bar—100 μm. **O,O´:** Ultrastructure of the dental stalk at stage E18. 1—loops of the basal lamina, 2—basement membrane, 3—mesenchymal cell, 4—glycogen, 5—mitochondria, 6—microvilli, 7—collagen fibrils, 8—cytoplasmatic process of mesenchymal cell. **P,P´,P´´,P´´´:** Ultrastructure of the dental stalk at stage P2. 1—intercellular spaces between epithelial cells, 2—microvilli of epithelial cells, 3—collagen fibrils, 4—loops of basement membrane, 5—mitochondria, 6—epithelial cells, 7—cytoplasmatic processes of fibroblast, 8—bundles of intermediate filaments. **Q,Q´,Q´´,Q´´´:** Ultrastructure of the dental stalk at stage P8. 1 - “folding” of lamina densa, 2—loops of basement membrane, 3—cytoplasmatic process of epithelial cell, 4—extracellular matrix, 5—collagen fibrils, 6—microvilli, 7—desmosome, 8—intermediate filaments. **R,R´,R´´,R´´´:** Ultrastructure of the dental stalk area at P14. 1—intercellular spaces between epithelial cells, 2—blood vessels, 3—nucleus of epithelial cell, 4—microvilli, 5—desmosome, 6—bundles of intermediate filaments, 7—keratohyaline granule, 8—degradated mitochondria

The first sign of a rudimental successional dental lamina initiation could be morphologically recognisable already at E16 in the area of the first lower molar when the tooth reached the early bell stage (Fig 2A). The RSDL appeared as a thickening on the lingual side of the outer enamel epithelium with increased numbers of PCNA-positive cells (Fig 2C). Later at E18, the RSDL expanded to form a short finger-like epithelial protrusion, which was clearly separated from the tooth germ at E18 (Fig 2D) and highly proliferative (Fig 2F). During these initiation and growth stages the RSDL was negative for cytokeratin (Fig 2B and 2E). During postnatal stages, the rudimental successional dental lamina became thinner and shorter in contrast to early stages (Fig 2J–2U), and it was more difficult to distinguish it. The RSDL was observed until stage P8 (Fig 2P), when only small finger like projections were seen at the base of the dental stalk. The RSDL was no longer observed at P12 and P14 (Fig 3I and 3K). The number of PCNA-positive cells decreased in the dental stalk as well as RSDL area during postnatal stages (Fig 2L and 2R, and S3 Fig) correlating with the decreasing size of this structure. On the other hand, as proliferation was reduced, the expression of cytokeratin was upregulated (Fig 2K and 2Q).

We then assessed the formation of RSDL using ultrastructural analysis (Fig 2G). The basement membrane of cells located at the tip of the RSDL at E18 exhibited foldings (Fig 2H). Epithelial cells in contact with the basement membrane had a cylindrical shape with oval nucleus and a smooth nuclear envelope (Fig 2H). These cells were in close contact with each other (Fig 2G). Mesenchymal cells surrounding this part of the lamina were flat and concentrically arranged around the finger-like protrusion (Fig 2G and 2I) with abundant macrophages (Fig 2I).

Ultrastructural analysis at P2 revealed several layers of epithelial cells contributing to the RSDL (Fig 2M). All cellular nuclei were still bordered by smooth and clear contours. The RSDL included a basal layer with high cylindrical shape and several layers of small polygonal cells in the middle (Fig 2M). Intercellular spaces were very minute with a small number of cytoplasmic processes (Fig 2M and 2N). The border with connective tissue was not straight and there were numerous folds and recesses with numerous lysosomes (Fig 2N and 2O).

At P8, the reducing RSDL contained only two basal layers surrounded by a basement membrane and thin central area morphologically consisting of one type of small cell (Fig 2S). All RSDL cells still exhibited a compact appearance with very small intercellular spaces (Fig 2S and 2T). Only apical cells contained smoothly bordered nuclei (Fig 2T). Nuclei of the other cells had irregular woven contours. All cells of the lamina possessed nuclei with signs of degradation (Fig 2U). Just a few organelles (mostly mitochondria) were located in the lamina cells corresponding to low cellular activity (Fig 2S and 2T). Only sporadic apoptotic cells were detected in the RSDL (data not shown).

### Morphology of the mouse dental stalk was modified during odontogenesis

At late embryonic stages, the tooth germ is connected to the oral epithelium by the dental stalk (Fig 3A). As the dental stalk is connected to the RSDL and undergoes remodelling before tooth eruption, we decided to focus also on the spatial and temporal changes in the stalk during this period. To observe disintegration of the dental stalk and to outline the epithelial cells, we performed immunostaining for cytokeratin from E18 to P16.

At E18, the dental stalk was formed from five to seven layers of epithelial cells with distinct buccal and lingual basal layers and a central core of less densely packed cells (Fig 3A and 3B). Outlines of the basal layer were not straight and there were sporadic thickenings forming protuberances of the epithelial layers. Cells in the basal layer of the oral epithelium were PCNA-positive, in agreement with the high proliferating activity of this region. Asymmetrical cell proliferation was observed in the dental stalk area with higher number of PCNA-positive cells on the lingual side of the dental stalk at E18 (Fig 2F and S3A Fig).

During postnatal stages (P0-P16), the dental stalk gradually thickened and became shorter (Fig 3C–3L). Furthermore, the oral epithelium above the dental stalk got thicker as keratinisation of superficial layers progressed and the number of layers increased (Fig 3K). The basal layer of the dental stalk became gradually corrugated. At later postnatal stages, the base of the epithelium was very rugged (Fig 3F, 3H and 3J). Bays of connective tissue protruded into the dental stalk epithelium at P8 (Fig 3F). Later, the continuity of epithelial layers was disrupted and small islands of epithelial cells dispersed in the mesenchyme were observed at P12 or P14 (Fig 3H and 3J). PCNA-positive cells were obvious in the basal layers of the dental stalk but the decrease in their number was statistically significant through development (Fig 2L and 2R, and S3D Fig).

The eruption of the first molar was preceded by a rupture of the oral epithelium covering the tooth and occurred after P14. Just before eruption, the dental stalk area was widely fenestrated above the erupting cusps and in the area in between them (Fig 3K and 3L). These bays contained highly vascularised connective tissue and numerous apoptotic bodies (Fig 3K and S4 Fig). At P16, different phases of eruption were seen in the anterior and posterior parts of the first molar (Fig 3M and 3N and S1 Fig). Dental stalk epithelium was still connected to the buccal part of the gingiva (Fig 3M). Degenerative processes also occurred in the remnants of dental stalk epithelium. Apoptosis, high vascularisation and keratinisation were characteristic for this area (S4 Fig). The buccal side of the dental stalk became part of the gingival epithelium (S5C and S5C´ Fig), while the most of the lingual cells died and tissue above the erupting tooth was shed into the oral cavity (S5D and S5E Fig).

### Dental stalk cells underwent differentiation and keratinization during postnatal stages

**Fig 4:**
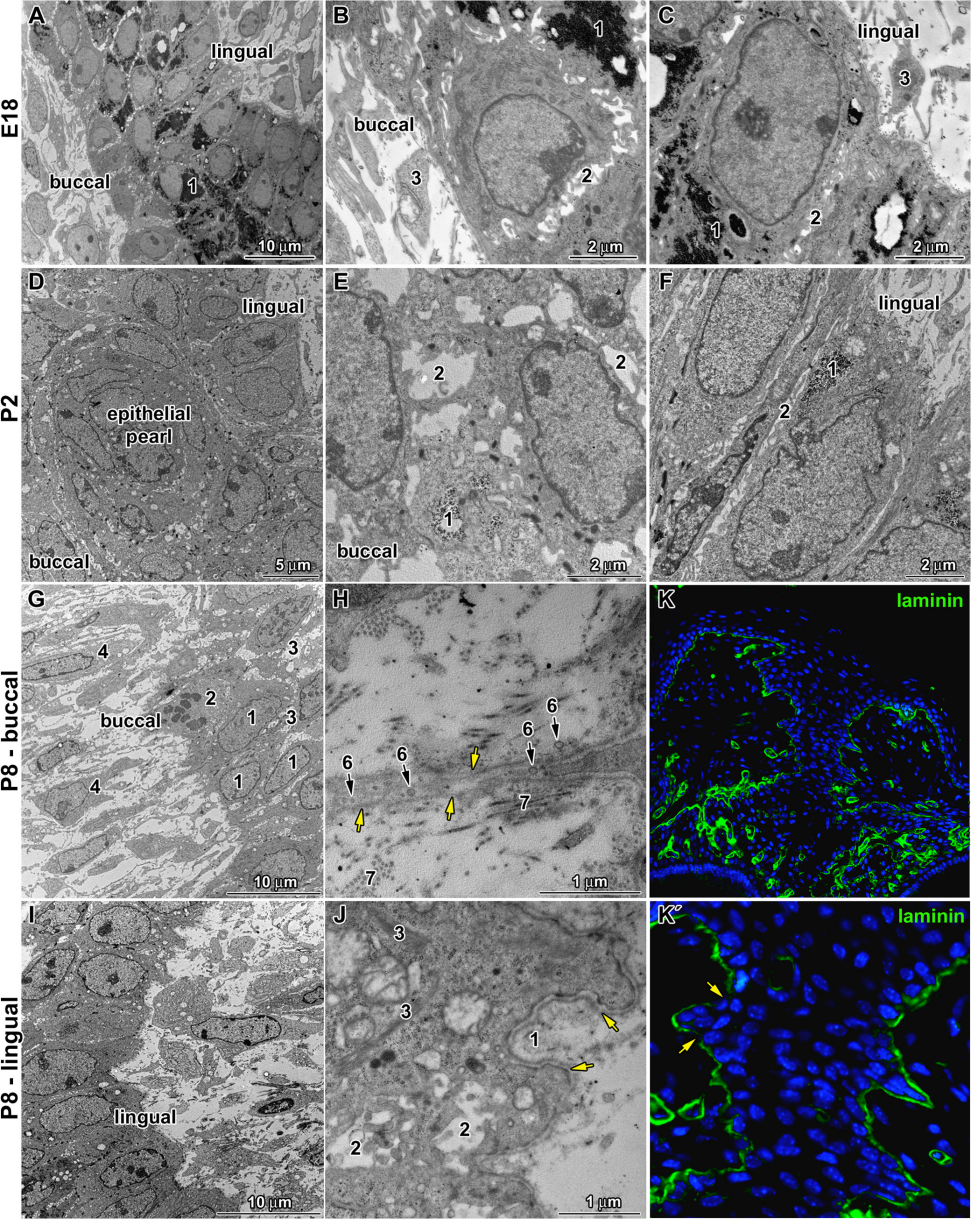
Differences between buccal and lingual parts of the dental stalk. **A:** Low power of the dental stalk area at E18. Central area contains large amount of glycogen (1). **B:** Detail of buccal side of the dental stalk at E18 with glycogen (1) in cytoplasm of deeper cells, expanded intercellular spaces (2) among epithelial cells and mesenchymal cells (3) in close proximity to basement membrane. **C:** Detail of lingual side of the dental stalk at E18 with high cylindrical cells separated by tight intercellular spaces (2) among epithelial cells. Large amount of glycogen (1) is located also in the basal layer of buccal cells. **D:** Low power of the dental stalk area at P2 with epithelial pearl in the central area. **E:** Detail of buccal side of the dental stalk at P2 with lower amount of glycogen (1) in the epithelial cells and extended intercellular spaces among them (2). **F:** Detail of lingual side of the dental stalk at P2 with reduced amount of glycogen (1) in contrast to previous stage as well as very tight intercellular spaces (2). **G:** Low power of the buccal side in the dental stalk area at P8. Epithelial cells (1) are separated by large intercellular spaces (3). Only occasional cells in mitosis (2) are observed. Long cytoplasmatic processes of fibroblasts (4) are oriented towards the epithelium. **H:** Detail of buccal side at P8, where epithelial cells send extremely long cytoplasmatic processes with numerous vesicles (6) out into the connective tissue. Basement membrane (yellow arrow) is surrounded by collagen fibers (7). **I:** Low power of the lingual side in the dental stalk area at P8. **J:** Higher detail of lingual side at P8 on basement membrane (yellow arrow) surrounded by large amount of extracellular matrix (1). Intercellular spaces (2) between epithelial cells contain numerous short cytoplasmatic processes. Bundles of intermediate filaments (3) are visible in the basal layer of the epithelium in the lingual side of the dental stalk. **K, K´:** Laminin labeling of the dental stalk area at P8 exhibited disruption of basement membrane (arrow) on the buccal side.

To further analyze morphological changes in the dental stalk area, we evaluated the stalk epithelium by transmission electron microscope. At E18, the dental stalk epithelium was found to interact with a well-defined basement membrane forming small loops (Fig 3O). Cells of the dental stalk contained numerous organelles including mitochondria and endoplasmic reticulum (Fig 3O´). Cells were glycogen-rich (Fig 3O), especially in the central area of the dental stalk (Fig 4A). Among several distinct types of intercellular connection between epithelial cells, desmosomes were prevalent. Abundant short cytoplasmic processes covered the surface of epithelial cells (Fig 3O´). Mesenchymal cells sent long cytoplasmic projections towards the epithelial cells (Fig 3O´). On its mesenchymal side, the basement membrane was in contact with a small amount of extracellular matrix with rare collagen fibres (Fig 3O´).

At P2, larger intercellular spaces were observed among epithelial cells (Fig 3P), which showed still few and short cellular processes (Fig 3P´). Cells contained numerous organelles including mitochondria and granular endoplasmic reticulum (Fig 3P´). Rare intermediate filaments formed only fine bundles in the epithelial cells (Fig 3P´´´). Larger duplications of lamina densa called "loops" were observed in several regions of the dental stalk (Fig 3P´). Long processes of fibroblasts were in contact with the basement membrane or fibroblasts sent out processes directly to bays among marginally located dental stalk cells (Fig 3P´´). Collagen fibrils in the surrounding connective tissue (Fig 3P´) were more frequent in comparison to embryonic stage (E18).

Later in development at P8, epithelial cells were connected by larger desmosomes when compared to the previous stage (Fig 3Q´´´). An increased number of microvilli was found on the cell surface and intercellular junctions between epithelial cells were observed (Fig 3Q´´´). Amount of intermediate filaments increased and formed large bundles in the epithelial cells (Fig 3Q´´´). “Folding” of lamina densa (Fig 3Q) or duplications of lamina densa called "loops" were observed in several regions of the dental stalk (Fig 3Q´). An enormous accumulation of amorphous extracellular matrix was observed in the area of cellular contact where the lamina densa became unclear (Fig 3Q´´).

Shortly before the eruption at P14, the epithelial cells were connected together by large desmosomes or nexuses (Fig 3R and 3R´). A large amount of elongated and branching microvilli filled the intercellular spaces and they were more frequent in comparison to earlier stages (Fig 3R´). There were degenerated mitochondria and long bundles of cytoplasmic filaments in dental stalk cells (Fig 3R´´ and 3R´´´). The nuclear envelope was irregular (Fig 3R´), and the cytoplasm was filled with intermediate filaments; organelles had vanished or were covered by filaments (Fig 3R´´´). Keratinisation of cells was observed in some areas of the dental stalk with keratohyaline granule accumulation, similar to the oral epithelium (Fig 3R´´´). The basement membrane was still present; however, morphology of the surrounding tissue was modified. Connective tissue with numerous capillaries penetrated into the dental stalk among epithelial cells (Fig 3R). Islands of connective tissue were separated from the epithelium by a basement membrane; however, capillaries did not possess their own basement membrane.

### Differences between the buccal and lingual sides of the dental stalk were evident on ultrastructural level

At E18, there were obvious morphological differences between lingual and buccal sides of the dental stalk as observed by electron microscope. The basal layer of lingual cells contained high cylindrical cells with oval nuclei, which were well organized (Fig 4C). They were closely attached to each other by numerous desmosomes and very limited intercellular spaces remained (Fig 4F). On the other hand, the buccal side contained smaller cells with round or flat nuclei and cells exhibited loose connections and large intercellular spaces (Fig 4B).

At postnatal stages, the lingual side of the dental stalk was usually less rugged and basal cells were arranged close to each other and seemed to firmly hold together with small extracellular spaces (Fig 4F). On the other hand, the arrangement of buccal cells seemed to be less organised (Fig 4E), more rugged and unrestricted with larger intercellular spaces filled by a rich extracellular matrix (Fig 4E). Some cells on the epithelium-mesenchyme border exhibited signs of migration, where some cells were observed to slide out of the lamina. Many epithelial cells of buccal side send extremely long and additionally branched cytoplasmic processes with numerous vesicles out into the connective tissue (Fig 4H). These processes were covered by the basement membrane (Fig 4H). Also, fibroblasts had long processes, which came into contact with the basement membrane (data not shown). Connective tissue around the dental stalk extended into several locations (e.g. macrophages were observed in this area); however, the basement membrane distinctly separated epithelial cells from fibroblasts (data not shown). On the lingual side, collagenous fibrils were not found in contact with the lamina densa (Fig 4J).

### Progenitor marker Sox2 was expressed in the dental stalk and rudimental successional dental lamina

Sox2 is one of the transcription factors expressed by putative tissue progenitor cells [20]. It was reported that Sox2 plays a role in maintaining cell pluripotency. In particular, it plays an essential role in controlling developmental processes in the early embryo. Sox2 was previously described to label the epithelial progenitor cells in mouse incisors [21], in the lamina cells that give rise to the sequential development of M1, M2 and M3 in the mouse [16], in the successional lamina and dental cord/stalk of the human primary molar, as well as in the reptilian dental lamina [16, 22]. It was proposed that Sox2 may play a role in the maintenance of the successional dental lamina, and Sox2 has been shown to be expressed in the lingual side of the dental stalk in mice at E16 and E16.5 [23]. In keeping with this, patients with mutations in *SOX2* exhibit dental anomalies including supernumerary teeth and the persistence of deciduous teeth [24]. Since the RSDL in mouse became reduced during postnatal stages, we hypothesised that temporal changes in Sox2 expression may cause progressive lamina regression.

**Fig 5:**
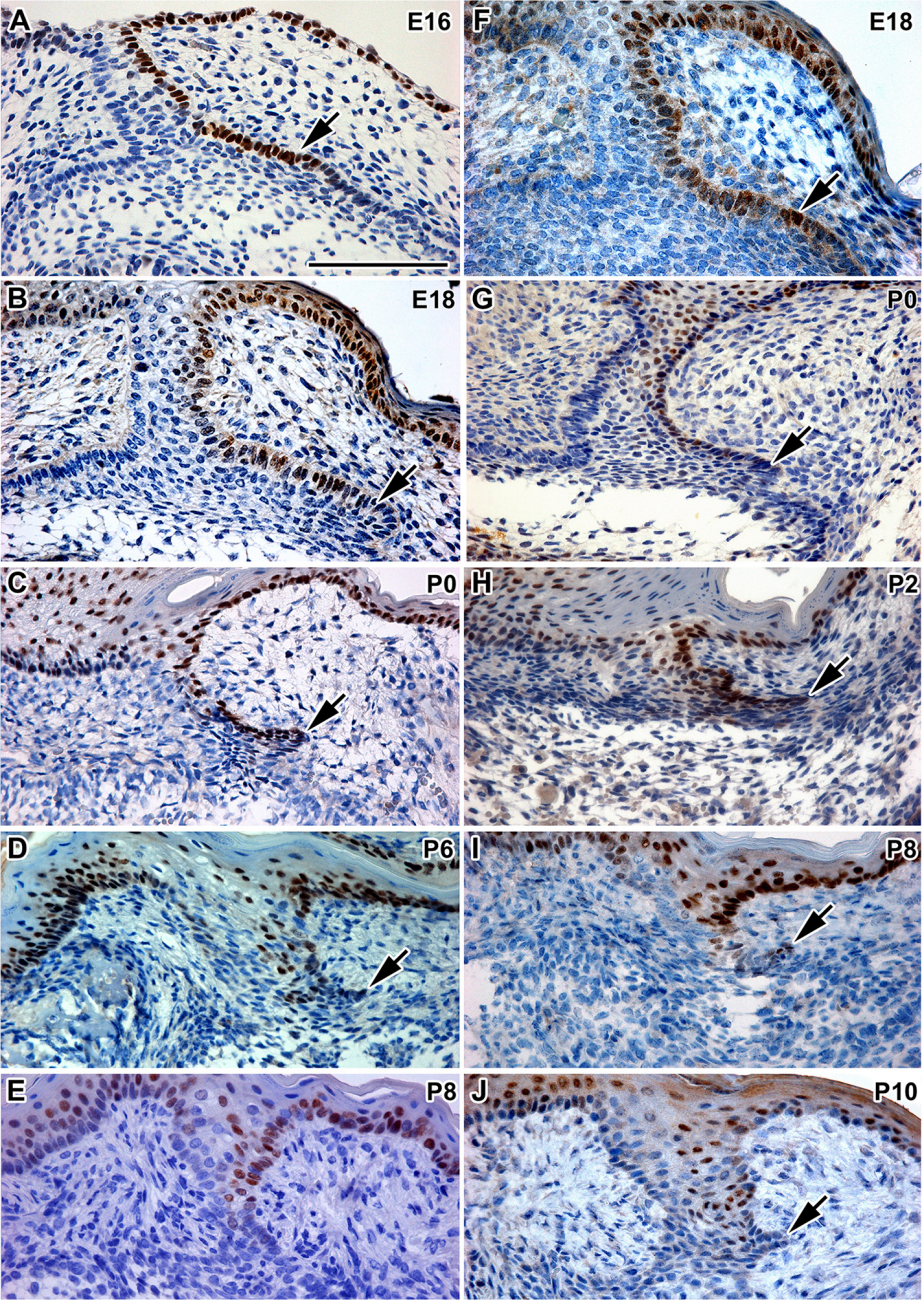
Sox2 protein expression in the dental stalk and rudimental successional dental lamina. **A:** The epithelial thickening (arrow) on the lingual side of the tooth bell of M1 is Sox2-positive at E16. **B**: During late embryonic stages, a rudimental successional lamina as well as lingual side of the dental stalk is Sox2- positive. **C, D:** At postnatal stages, Sox2 is downregulated in the rudimental successional dental lamina of M1. **E:** At P8, the amount of Sox2-positive cells is decreased in the rudimental successional dental lamina. **F:** The epithelial thickening on the lingual side of the tooth bell of M2 is Sox2-positive at E18. **G,H:** Rudimental successional lamina is Sox2- positive at early postnatal stages (P0 and P2). **I:** Expression of Sox2 is downregulated in the rudimental successional dental lamina of M2 at P8. **J:** At P10, the amount of Sox2-positive cells is decreased in the dental stalk of M2. Sox2-positive cells are labeled by DAB (brown nuclei). Negative cells are counterstained by Hematoxylin (blue nuclei). Scale bar—100 μm

In our study, we used immunohistochemical detection to analyse Sox2 protein expression in the successional dental lamina as well as the dental stalk during embryonic and postnatal stages. At prenatal stages, Sox2 expression was asymmetrical in the dental tissue of the first molar. At the early bell stage (E16), Sox2 expression was observed in the basal layer of the lingual side of the dental stalk and in the thickening future rudimental successional dental lamina, which was forming in the close proximity of the outer enamel epithelium (Fig 5A). When rudimental dental lamina was distinct (E18), Sox2 was expressed again on lingual side of the dental stalk and more superficial part of the rudimental successional dental lamina (Fig 5B). This pattern of Sox2 expression also remained during the early postnatal stages (P0, Fig 5C). Later in development (P6), the successional lamina was reduced in size and Sox2 expression was gradually down-regulated (Fig 5D). At stage P8, Sox2 expression almost disappeared and only dispersed positive cells were observed in the dental stalk area (Fig 5E). Interestingly, similar asymmetrical bucco-lingual expression was observed in the basal cells of the oral epithelium adjacent to the dental stalk, where stronger expression was located in the lingual side and no expression was seen in the buccal area (Fig 5E). Same Sox2 expression pattern was observed in the dental stalk and RSDL of the second molar (Fig 5F–5J) only with time delay.

These results indicate that the lingual side of the mouse dental stalk and the oral epithelium consisted of cells with pluripotent capability, which was lost during development at the postnatal stages. Our results support the hypothesis that loss of Sox2 signal in the successional dental lamina could play a role in the failure of replacement tooth initiation in monophyodont mouse as progenitor cells are deficient.

## Discussion

There are significant variations in successional dental lamina morphology and development in monophyodont, diphyodont and polyphyodont species. However, detailed processes establishing the differences in early dental development that result in species-specific setup of tooth generation number are unknown. The mouse, as the main model for tooth developmental study, forms only one tooth generation. The main aim of this paper was to reveal the morphological changes in the successional dental lamina and the dental stalk during development in the mouse, since they potentially underly monophyodonty. The focus of our paper was on initiation and degeneration of the rudimental successional dental lamina, as it reduces in size, decreases the number of epithelial layers and finally disappears during development.

### A rudimental epithelial protrusion similar to successional dental lamina is initiated in monophyodont species

Mammals lost replacement teeth for molars more than 200 million years ago [25]. However, here, our study showed the initiation and long persistence of the rudimental epithelial thickening on the lingual side of the mouse dental stalk similar as was previously observed in the rat [18], bearded dragon [19, 26] and chameleon [13]. In these species, rudimental finger-like protrusion are also initiated and their size decreases during post-hatching development without initiating replacement teeth [13]. Based on identical morphology and specific location on the lingual side, which is equivalent to the successional dental lamina in non-mammalian species as well as diphyodont mammals, we propose this structure as the rudimental successional dental lamina. Moreover, the timing of initiation of this epithelial protrusion was same as in the case of replacement lamina of premolars in diphyodont dentition. However, experimental proof of its tooth replacement capacity is still necessary.

In monophyodont rats, the initiation of a secondary tooth bud has been described [18]; however, careful analysis of the pictures revealed only similar rudimental structures as observed here in mouse with the initiation of the successional lamina. Moreover, replacement teeth never formed. The rat rudimental successional lamina was observed on the lingual side of the molar at stage P0 and P5 but it was no longer visible at P10 [18]. In the mouse, the rudimental successional lamina was visible for a much longer period: from E16 to P10.

While species with one tooth generation can initiate successional dental lamina, the potential to form replacement teeth is inhibited during odontogenesis and the successional dental lamina regresses. To explore possible developmental processes contributing to rudimental successional dental lamina regression, we focused on the stages just before birth, up to the eruption of the first molar. We found that the RSDL passes through morphological remodelling, as observed at histological and ultrastructural levels.

In the mouse, there was neither fragmentation of the successional dental lamina nor the formation of epithelial islands in contrast to what has been reported in diphyodont species after the initiation of the replacement tooth [10–12]. Instead, there was a morphological aspects of successional lamina regression in the mouse appeared similar to what was reported in other monophyodont animals, showing a gradual reduction in size during postnatal/post-hatching stages [13]. We also observed evident changes in the ultrastructure of the murine RSDL lamina during development. A small number of cytoplasmic processes together with few desmosomes were found in contrast to the dental stalk area. A decrease in the number of organelles corresponded to the low metabolic activity of these cells at late postnatal stages. The number of cell layers decreased, also during postnatal stages, and the shape of the nuclei were modified with signs of degradation. Macrophages were located around the tip of the rudimental successional dental lamina in mouse, where numerous folds were located.

Several mechanisms can contribute in the reduction of the successional dental lamina in monophyodont species, including cellular senescence, increased apoptosis, decrease in proliferation, migration of cells out of the lamina, and loss of progenitor cells.

Massive apoptosis was not observed in the mouse RSDL similarly as was previously described in the chameleon [13]. However, proliferation decreased significantly during postnatal stages particularly on the lingual side of the successional dental lamina. These results suggest that the low rate of proliferation is probably not able to support the growth of this lamina and its maintenance in the mouse.

### Possible role of *Sox2* during odontogenesis

Recent studies have focused on the location of possible sources of odontogenic progenitor cells for tooth renewal [6, 19, 27]. As replacement dental tooth germs is initiated from the successional dental lamina, putative progenitor markers such as *Lgr5* and *Sox2* have been analysed in this area in diphyodont species [16]. Furthermore, the distribution of BrdU label-retaining cells allowed the identification of slow cycling stem cells in alligator and gecko dental laminae [28, 29].

The transcription factor *Sox2* is involved in the determination of cell fate in neural and epithelial lineages [21, 30]. Furthermore, *Sox2*-positive cells were found to be important for progenitor survival during tissue regeneration [31]. Patients with mutations in *Sox2* exhibit dental anomalies including supernumerary teeth and the persistence of deciduous teeth [24]. Conditional *Sox2* KO-mice exhibited the early formation of hypoplastic dental stalk [16]. Similarly, we observed down-regulation of *Sox2* expression, followed by later hypoplasia of the dental stalk during late postnatal stages.

It was previously proposed that *Sox2* may inhibit the proliferation of progenitor cells in the dental lamina and maintain these cells as odontogenic progenitors [16]. However, our study, showed a higher number of proliferative cells on the lingual side of the dental stalk, where *Sox2*-positive cells were also located. Furthermore, a decrease of proliferation on the lingual side occurred simultaneously with down-regulation of *Sox2* expression. Since experimental overexpression of *Sox2* in mesenchymal progenitor cells derived from bone marrow resulted in higher proliferation and differentiation [32], we propose that *Sox2* expression on the lingual side is necessary to maintain progenitor cell population proliferation in the successional dental lamina. To support our hypothesis, future work will be necessary to analyse *Sox2* expression patterns in other monophyodont species and to design functional experiments.

### The different fate of the dental stalk and the successional dental lamina in mouse

During the postnatal period, continuous morphological changes occurred within the dental stalk of the mouse molar. In contrast to the successional dental lamina, epithelial integrity of the dental stalk was progressively disrupted in the mouse. Just before eruption, the dental stalk area was widely fenestrated above and in between the erupting cusps. Bays of highly vascularised connective tissue protruded into the epithelium. The extracellular matrix, which filled the bays, was probably produced by fibroblasts and altered metabolism of nearby epithelial cells. The numerous lysosome-like inclusions in fibroblasts suggested that these cells could cause disruption of the basement membrane in contact with the dental lamina. Similar connective tissue growth through the epithelium was previously observed in the rat [18]. Bays filled with fibroblasts appeared during the late bell stage of the enamel organ in the rat, while we observed them later during development, at P8, in the mouse. Cells adjacent to the dental stalk showed no sign of cell death linked with bay formation.

Previously published data [33, 34] suggest that apoptosis might play a role in late embryonic stages during successional dental lamina regression or dental stalk degradation. However, only rare apoptotic bodies were observed in the successional dental lamina throughout development. Apoptosis thus does not play a major role in the reduction of the successional dental lamina. On the other hand, there was an increase of apoptotic bodies in the superficial areas of the dental stalk in close proximity to the oral epithelium during late postnatal stages (P14), just before the first molar erupted. This high level of apoptosis meant that only cells on the buccal side of the dental stalk became part of the dental gingiva.

**Fig 6:**
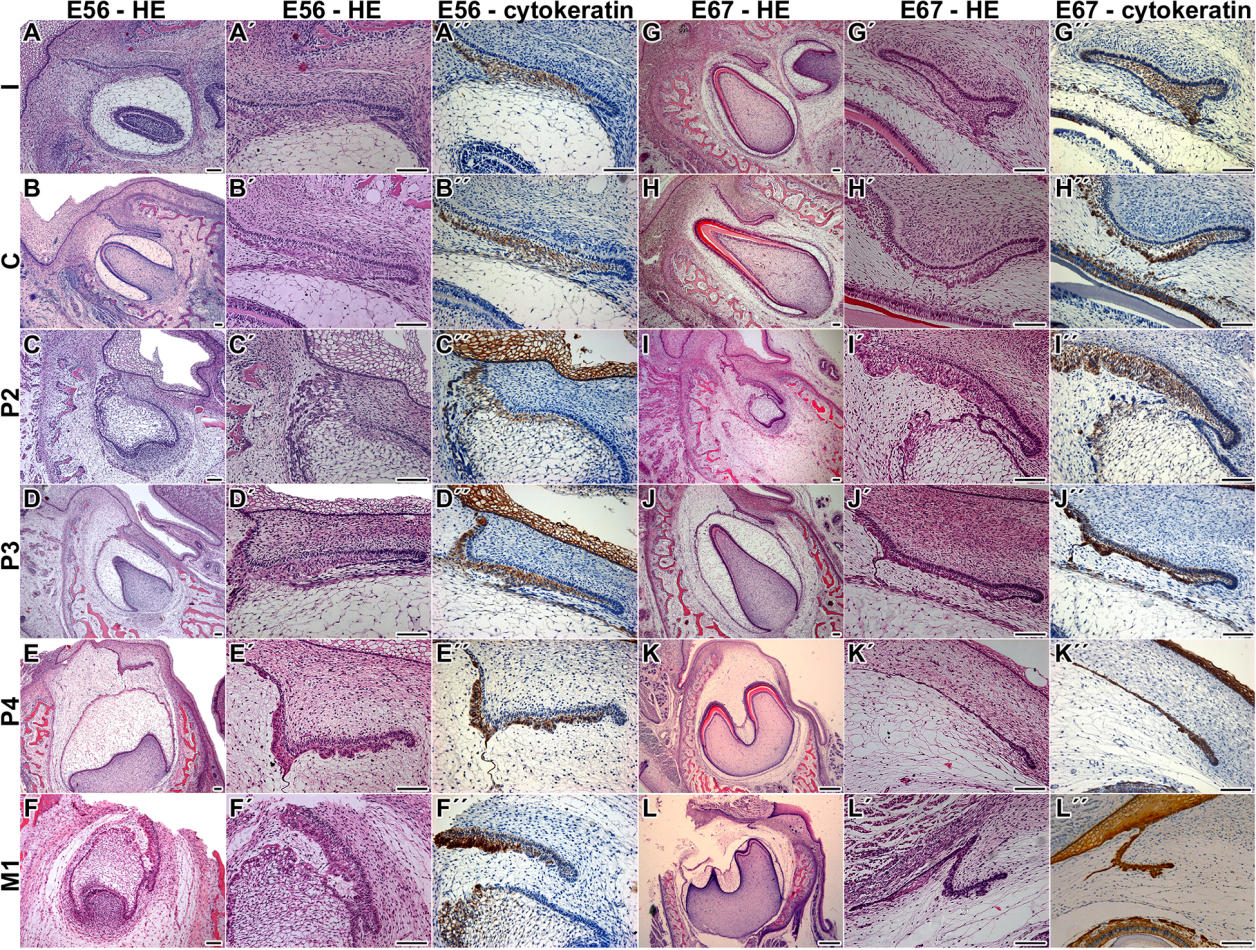
Successional dental lamina in pig embryos. Cytokeratin-positive cells are labeled by DAB (brown cells). Negative cells are counterstained by Hematoxylin (blue nuclei). Scale bar—100 μm

The process of dental stalk degradation in mouse is different to that which can be observed in diphyodont species such as ferret, pigs or hedgehog (Fig 6, S6 Fig), where the dental lamina is disconnected from the oral epithelium and fragmented into parts [11, 12, 35]. Furthermore, cells facing the tooth anlagen enlarged cytoplasmic processes (Fig 6 and S6 Fig) and some were disconnected from the lamina [12]. However, the mouse dental stalk remained connected to the oral epithelium up to tooth eruption. The epithelial cells formed very long cytoplasmatic processes sticking out into the mesenchyme or surrounding the connective tissue during postnatal stages. Therefore, the morphological changes during dental stalk degradation in the mouse are different from those described in diphyodont dentition.

Surprisingly, distribution of desmosomes exhibited changes during postnatal stages, where more and larger desmosomes were visible among epithelial cells at later stages. As we observed a larger dental stalk area at late postnatal stages and bays of connective tissues, we expected the opposite effect with loosening connections among epithelial cells. This is probably related to epithelial cells’ keratinisation in the dental stalk area. Similar increases in desmosomal connections among cells were observed during the differentiation of keratinocytes in the skin, while basal layers contained only a small number of desmosomes [36]. The increase in number of desmosomes together with the accumulation of keratin was also observed in hair cells [37]. The formation of these junctional complexes is important for the cohesion of cuticle cells. These modified large desmosomes, also known as corneodesmosomes, arise from the extension of several desmosomes by their connection together with keratin accumulation [38].

Furthermore, we observed nuclear degradation, the removal of organelles and the accumulation of intermediate filaments, which were also connected to epithelial keratinisation. Keratohyaline granules were observed in the dental stalk area at older stages. As lateral parts of the dental stalk became part of the gingiva, keratinisation of cells is critical for the formation of a firm envelope.

### What distinguishes the lingual from the buccal side of the dental stalk area?

The tooth germ was initiated close to the oral epithelium in mouse and the enamel organ of the first molar was connected to the superficial epithelium up to tooth eruption by the dental stalk. Differences exist between the lingual and buccal sides of the dental stalk, which can be observed at histological, ultrastructural and molecular levels. The shapes of basal cells and their nuclei were different with cylindrical nuclei on the lingual side and more condensed round nuclei on the buccal side. Later in development at P8, buccal epithelial cells formed long cytoplasmic processes sticking out into the connective tissue and loose connections between these cells were observed on the buccal side. Similar formation of elongated cytoplasmatic epithelial processes also occurred on the buccal side of the pig dental lamina [12]. However, in the pig, epithelial cells lost the contact with the dental lamina and formed clusters in the close proximity of the lamina (Fig 6). Furthermore, small blood vessels started to appear at late embryonic stages in pig just next to the acidophilic cells. In mouse, we also observed close contact of blood vessels with the dental stalk. In mouse, there was no side-specific appearance of vessels as they progressed into the epithelium from both sides of the dental stalk. Based on our results, there seems to be a close temporal and spatial relationship between the initiation of angiogenesis in adjacent mesenchyme and disruption of the dental stalk in the mouse. The entrance of vessels was probably enabled by structural modifications of the basement membrane at later postnatal stages as showed by laminin labeling. The exact function of capillaries in the dental stalk is unknown; however, they can bring macrophage-like cells, which can contribute to the degradation of extracellular matrix and cytokine secretion [39].

A large amount of extracellular matrix was observed around the basement membrane on the buccal side of the dental stalk at later stages of odontogenesis. Similarly, the increased amount of fibrillar structures associated with the basement membrane was observed in the area between odontoblasts and the inner enamel epithelium [40]. The basement membrane and associated material might contribute to the epithelial-mesenchymal interaction through the extracellular matrix surrounding the dental stalk, as this matrix can serve as a pool of paracrine factors such are BMPs and FGFs, which bind to extracellular proteins.

The basement membrane of the dental stalk formed loops and multiple superposed layers similar to that previously described on the buccal side of mouse incisor [41]. Although the basement membrane plays an important role in the control of cell differentiation [42, 43], the presence of multiple superposed basement membranes in cultured incisors had no consequence on odontoblast differentiation [44]. We observed these modifications of the basement membrane in the dental stalk area, where fibroblast processes were in contact with epithelial cells. Interestingly, similar modifications of the basement membrane with local detachments, loops and duplications of the basement membrane were observed during inter-cusp folding (at E16 and E18) in the mouse molar [45, 46]. It was proposed that specific changes in the basement membrane associated with the enamel knot enable the segregation of these cells and their migration [46]. The morphological changes in basement membrane observed in the dental stalk, might thus reflect cell rearrangement in the epithelium. However, in the future, it will be necessary to obtain experimental proof of whether some epithelial cells might migrate to neighbouring connective tissue and undergo epithelial-mesenchymal transformation as in the pig dental lamina [12].

Furthermore, differential proliferation was found on both sides of the dental stalk with a higher number of PCNA-positive cells on the lingual side at E18. This is in agreement with similar buccal-lingual differences observed in other species [47]. Asymmetrical proliferation in the dental lamina was previously proven to be connected with the asymmetrical expression of *Shh*, which can stimulate proliferation [48, 49]. *Shh* loss-of-function experiments altered the proliferation pattern in the python dental lamina [7]. Moreover in the snake, *Shh* expression was found in the oral-dental interface, specifically on one side of the lamina, directing growth of the dental lamina [7]. However, *Shh* expression is not present in the oral-dental interface in mouse [50], which can be one reason for the straight growth of dental lamina into the underlying mesenchyme.

### Future directions

From previous morphological and developmental comparisons, there is an obvious link between the formation of large and persisting dental lamina in Amniotes and a higher number of replacement teeth. It seems that the early regression of successional dental lamina in mammals leads to a decrease in the number of replacement tooth generations. Furthermore, there are species-specific differences in the mechanism of dental lamina degradation in relation to the different number of tooth generations. However, many questions still remain. For example, what regulates the switch between maintenance and regression of the successional dental lamina? Also, what initiates dental lamina degradation? More vertebrate species will have to be analysed to make more general conclusions.

In summary, the uncovering of detailed processes during successional dental lamina degradation can help to clarify the evolutionary routes utilised to control number of tooth generations in mammals, including humans.

## Supporting Information

S1 Fig Dental stalk morphology in the transitional areas among teeth. Scale bar—100 μm
(TIF)

S2 Fig Rudimental successional lamina in the upper jaw of mouse. **A, A´:** Small epithelial protrusion is visible on the lingual side of the first molar in the upper jaw at E18. **B, B´:** Thin successional lamina is still visible at P4. Scale bar—100 μm
(TIF)

S3 Fig Analysis of PCNA-positive cells in the dental stalk area. **A-C:** Area of the dental stalk, which was selected for the analysis, is outlined. **D:** The number of PCNA-positive cells was decreased in time and differences among stages were statistically significant. Results are displayed as proliferating index (number of positive cells/ total number of cells). PCNA-positive cells are labeled by DAB (brown nuclei). Negative cells are counterstained by Hematoxylin (blue nuclei). (ANOVA, * *p*<0.05, ** *p*<0.01, *** *p*<0.001). Scale bar—100 μm
(TIF)

S4 Fig Apoptosis in the dental lamina of mouse during late postnatal stages. Apoptotic cells were detected by TUNEL (brown nuclei, arrows). Negative cells were counterstained by Hematoxylin (blue nuclei). TUNEL-positive cells are rare at early developmental stages but their number increases just before the tooth eruption. Scale bar—100 μm.
(TIF)

S5 Fig Disintegration of dental lamina at P16 in mouse. **A-E:** Transversal sections through the first molar in anterior to posterior sequence exhibit different stages of tooth eruption with more progress in the anterior area **(A)** and the eruption just occurring in the posterior area **(E)**. Scale bar—100 μm
(TIF)

S6 Fig Dental lamina morphology in the lower jaw of neonatal hedgehog. sdl—successional dental lamina, st—successional tooth anlagen. Scale bar—100 μm.
(TIF)
