# Supplementary figures and images for: Fate of the Molar Dental Lamina in the Monophyodont Mouse
Source: PLoS One. 2015 May 26;10(5):e0127543. doi: 10.1371/journal.pone.0127543 (PMC4444311; doi:10.1371/journal.pone.0127543)

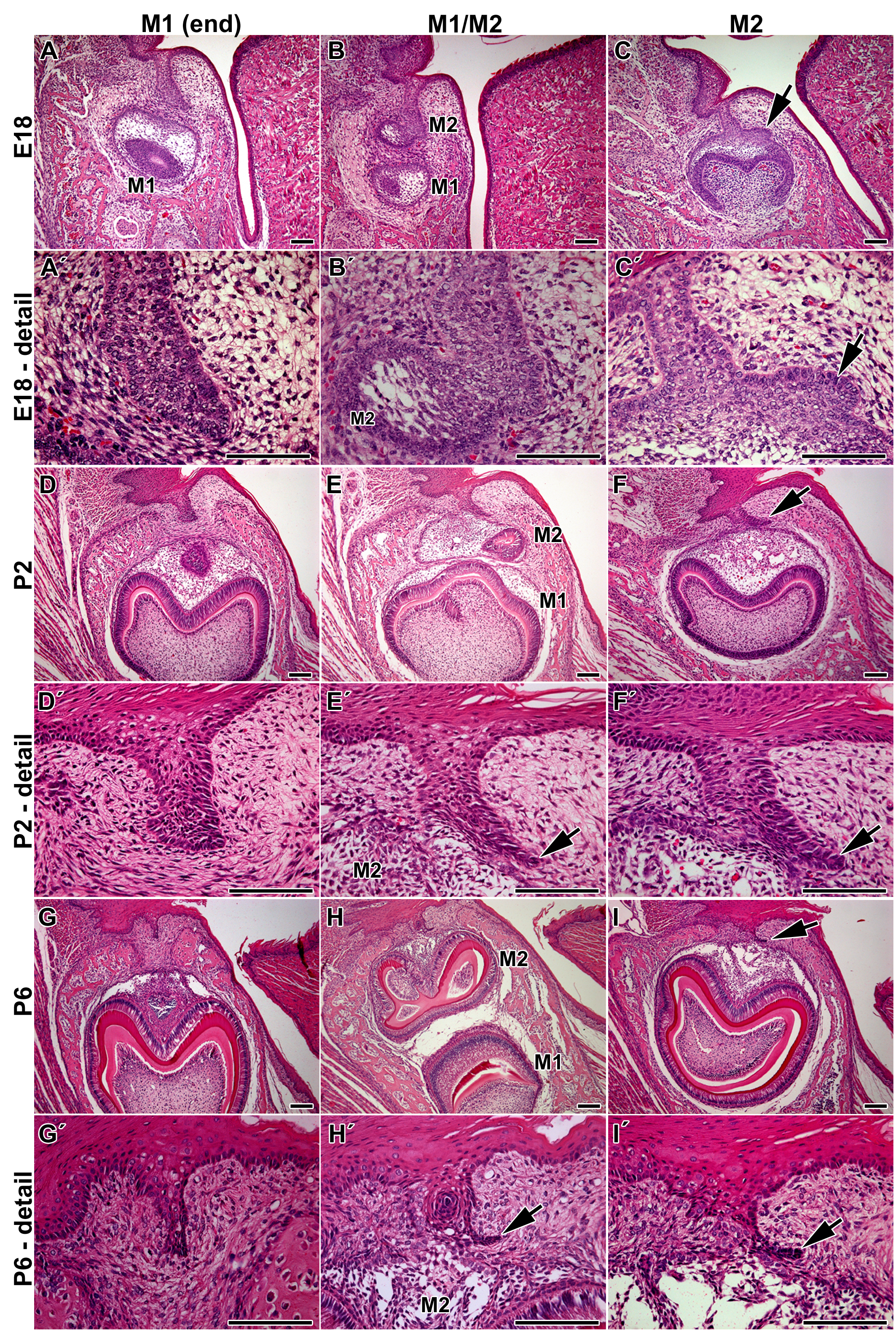

Supplement: S1 Fig — Scale bar—100 μm (TIF) [file pone.0127543.s001.tif]

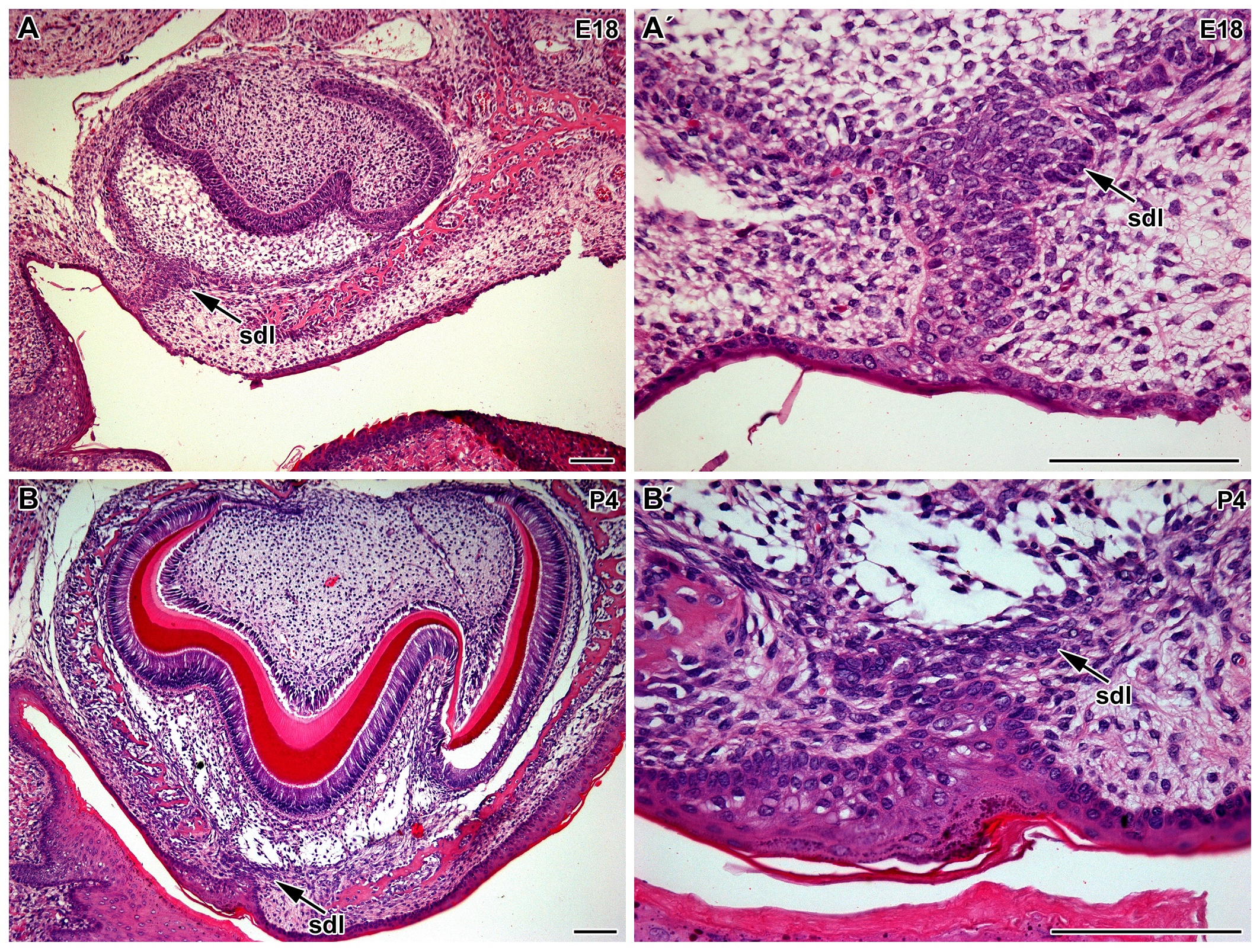

Supplement: S2 Fig — A, A´: Small epithelial protrusion is visible on the lingual side of the first molar in the upper jaw at E18. B, B´: Thin successional lamina is still visible at P4. Scale bar—100 μm (TIF) [file pone.0127543.s002.tif]

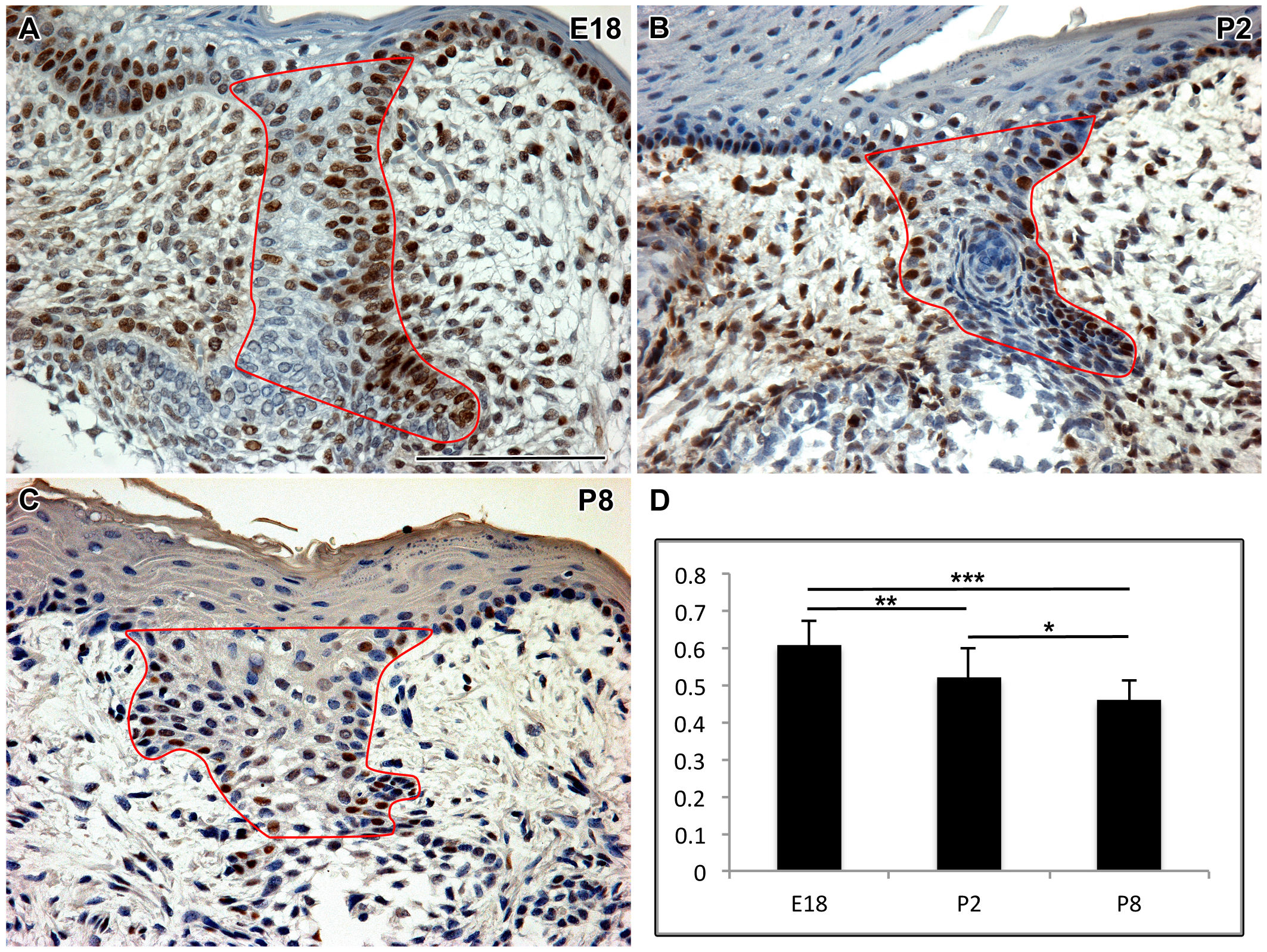

Supplement: S3 Fig — A-C: Area of the dental stalk, which was selected for the analysis, is outlined. D: The number of PCNA-positive cells was decreased in time and differences among stages were statistically significant. Results are displayed as proliferating index (number of positive cells/ total number of cells). PCNA-positive cells are labeled by DAB (brown nuclei). Negative cells are counterstained by Hematoxylin (blue nuclei). (ANOVA, * p<0.05, ** p<0.01, *** p<0.001). Scale bar—100 μm (TIF) [file pone.0127543.s003.tif]

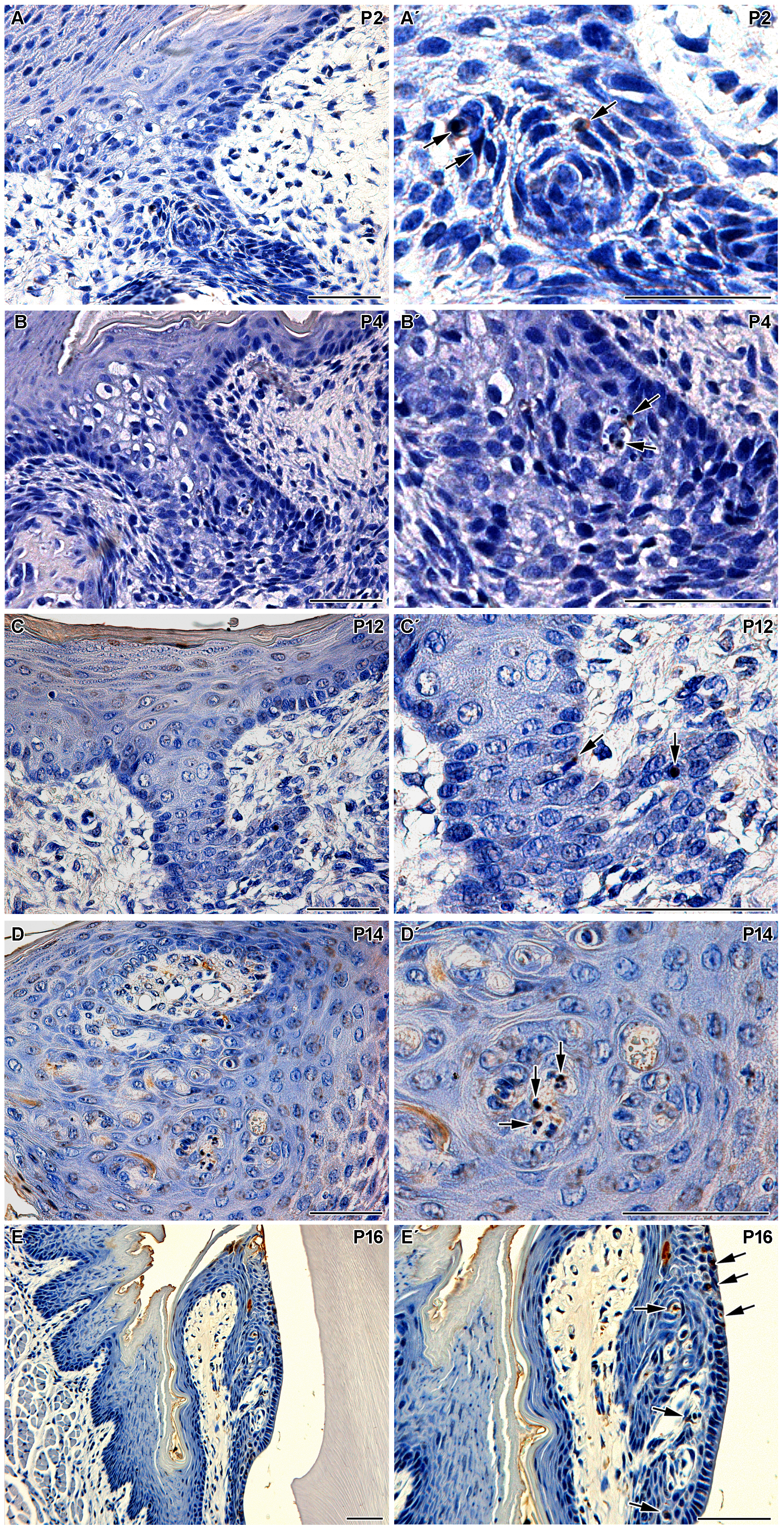

Supplement: S4 Fig — Apoptotic cells were detected by TUNEL (brown nuclei, arrows). Negative cells were counterstained by Hematoxylin (blue nuclei). TUNEL-positive cells are rare at early developmental stages but their number increases just before the tooth eruption. Scale bar—100 μm. (TIF) [file pone.0127543.s004.tif]

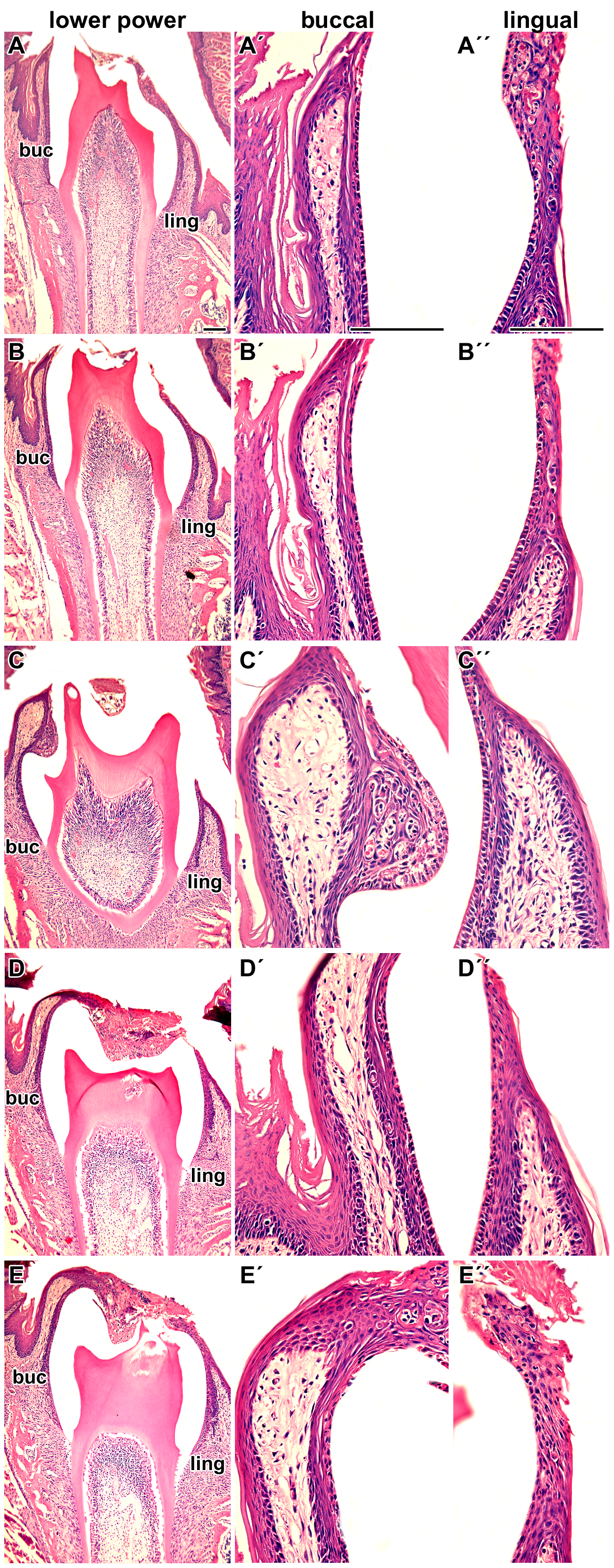

Supplement: S5 Fig — A-E: Transversal sections through the first molar in anterior to posterior sequence exhibit different stages of tooth eruption with more progress in the anterior area (A) and the eruption just occurring in the posterior area (E). Scale bar—100 μm (TIF) [file pone.0127543.s005.tif]

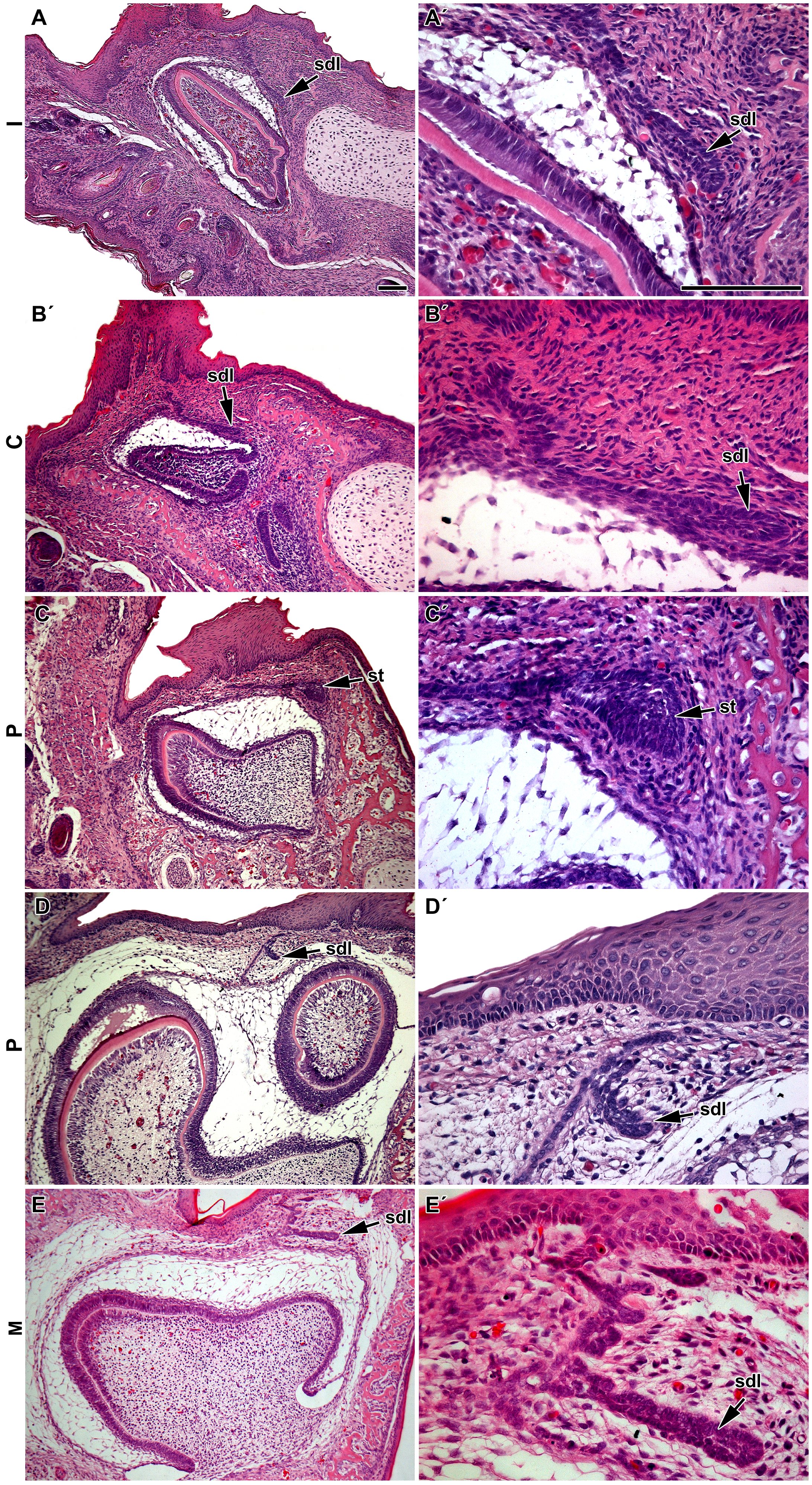

Supplement: S6 Fig — sdl—successional dental lamina, st—successional tooth anlagen. Scale bar—100 μm. (TIF) [file pone.0127543.s006.tif]
